# Genomes, structural biology and drug discovery: combating the impacts of mutations in genetic disease and antibiotic resistance

**DOI:** 10.1042/BST20160422

**Published:** 2017-04-13

**Authors:** Arun Prasad Pandurangan, David B. Ascher, Sherine E. Thomas, Tom L. Blundell

**Affiliations:** Department of Biochemistry, University of Cambridge, Tennis Court Road, Cambridge, U.K.

**Keywords:** antimicrobial resistance, genetic disease, mutational analysis, structure-guided drug discovery

## Abstract

For over four decades structural biology has been used to understand the mechanisms of disease, and structure-guided approaches have demonstrated clearly that they can contribute to many aspects of early drug discovery, both computationally and experimentally. Structure can also inform our understanding of impacts of mutations in human genetic diseases and drug resistance in cancers and infectious diseases. We discuss the ways that structural insights might be useful in both repurposing off-licence drugs and guide the design of new molecules that might be less susceptible to drug resistance in the future.

## Structure-guided drug discovery

Ideas about the use of protein structure in drug discovery emerged in parallel with the first three-dimensional structures defined by X-ray crystallography. An early focus on the structure of haemoglobin in the 1950s stimulated thoughts about sickle cell disease in the Perutz laboratory in Cambridge [1,2], and the structure of lysozyme in 1966 in the Phillips' laboratory at the Royal Institution in London described a well-defined active-site cleft, interactions with substrate and early hypotheses about ligand binding and mechanism [3]. The structure of insulin in 1969 [4] was already a subject of interest in the pharma industry as insulin crystals were used to treat diabetics. The many sequences of insulin, already defined by Sanger in Cambridge, together with efforts to synthesize the protein underway in New York, Aachen and Shanghai, led to thoughts about design of more effective and longer-lasting insulins for therapeutic use [5]. The availability of further sequences in the years following led to early attempts to model human α-lactalbumin, a homologue of lysozyme found in milk [6,7], and then later relaxin [4] and insulin-like growth factor [8], both distant homologues of insulin of interest in biomedicine.

Although some laboratories, such as that of Dorothy Hodgkin, were well linked in to the pharma industry, interest elsewhere in structure-guided drug discovery increased only as experimental structures of enzymes and their complexes became available in academia, often for paralogues of current drug targets in industry. An example of this trend was renin, which had been known for many years to be involved in regulating blood pressure through its role in processing angiotensinogen to angiotensin I [9]. Aspartic proteinase structures of paralogues of renin had been defined in the 1970s, and these allowed models to be created for renin, for example, by Sibanda and colleagues [10]. These were used in some of the earlier structure-guided drug discovery campaigns for antihypertensives. The X-ray structures of complexes of renin inhibitors complexed with paralogues of renin appeared later [11] and were eventually followed by high-resolution structures of renins [12,13].

## Chemical library screening strategies exploiting structure

These developments in structure-guided drug discovery occurred in parallel with an increasing interest in target-agnostic phenotypic screening as well as roboticised screening of individual targets with chemical libraries of increasing size. However, for drug-like molecules (optimally of molecular mass ∼500 Da), screening requires libraries much larger and more diverse than the ∼10^6^ molecules available to most large pharma companies. The urgent need for a more innovative approach was underlined by the exponential increase in costs in drug discovery research and development in the 1990s, but the relatively low output of new drug approvals. Two innovative approaches involving protein structures that have provided complementary strategies have been virtual screening of very large numbers of compounds and experimental fragment-based drug discovery.

Virtual screening explores possible binding sites *in silico* for a large number of small molecules [14–17], assessing electrostatic, van der Waals or hydrogen-bonding interactions involved in molecular recognition of candidate molecules for binding a target of known structure. Virtual screening methods (such as GOLD [18], AUTODOCK [19] and GLIDE [20]) are used to dock large and diverse sets of drug-like molecules, in order to identify compounds that might provide useful ‘hits'.

In contrast, fragment-based drug design is mainly an experimental approach to the challenge of moving from hits to leads. It depends on decreasing size and complexity by exploiting a fragment library, reduced in molecular mass (<300 Da) and in number to around a thousand molecules, which are screened against a target of interest using biochemical, biophysical and structural methods [21,22]. The fragments are developed into lead candidates by chemically growing or linking the fragments, thereby exploring the chemical space available for binding to the target protein very effectively. Although low-molecular-weight fragments have relatively lower potency than the more complex molecules found in typical high-throughput screening compound libraries, small fragments that bind, do so by making well-defined and high-quality interactions and by displacing ‘unhappy' water molecules at hotspots on the protein [23,24].

At first fragment-based drug discovery was focused on ‘druggable' targets with large, well-defined cavities, such as protein kinases; it was pioneered both in large companies such as Abbott, who used SAR by NMR (structure–activity relationships by nuclear magnetic resonance (NMR) [25]), as well as in small start-ups such as Astex, which has focused on high-throughput X-ray crystallography to screen fragments [21]. Increasingly now fragment-based approaches involve, first, a range of biophysical methods such as surface plasmon resonance (SPR) and thermal shift to screen a fragment library, and, second, others to provide a detailed analysis of the three-dimensional structure of the fragment complex by X-ray crystallography or NMR, the thermodynamics by isothermal calorimetry and kinetics by SPR. Molecular dynamics can also be used to explore different conformers of the protein and even reveal cryptic sites.

Until Otsuka purchased it in 2013, Astex brought compounds into clinical trials within the company, but also importantly developed strategic alliances with larger companies, including Jannsen, Novartis, AstraZeneca and GlaxoSmithKline. Astex has made impressive progress in clinical trials, achieving a milestone on 1 November 2016 with US FDA's filing of a new drug application for LEE011 (ribociclib), a drug that targets protein kinase CDK4, which was developed in an alliance with Novartis; this will be used in combination therapy with letrozole as a first-line treatment for advanced breast cancer.

In academia there have been attempts to use fragment-based drug discovery to target sites that have been previously defined as ‘undruggable', most often interfacial or allosteric sites [26]. An example of this has been the use of fragment-based drug design to target the binding site of BRCA2-BRC repeats on Rad51, which catalyses an ATP-dependent DNA strand exchange in repair by homologous recombination of DNA double-strand breaks. This interaction involves concerted folding and binding of the BRC repeat, a foldable amino acid sequence within intrinsically disordered regions of BRCA2, onto the globular structure of Rad51 [27]. The BRCA2 interacts first through docking of a phenylalanine within a conserved FXXA sequence into a well-defined pocket of Rad51. This provides an anchor for the subsequent folding as a β-turn of the BRC repeat sequence, in order to allow the conserved alanine to bind into a smaller, more hydrophobic pocket. A further interaction is formed by the folding as an α-helix of a C-terminal region of the repeat motif into a shallow binding cleft. This was proposed as a possible site for targeting inhibitors and required very different chemistry from the ‘drug-like’ molecules designed to bind classical targets like protein kinases. The small pockets are well suited to bind fragments [28] and Cambridge labs in Biochemistry, Chemistry and Oncology have subsequently developed nanomolar inhibitors to bind this site [29].

**Figure 1.**
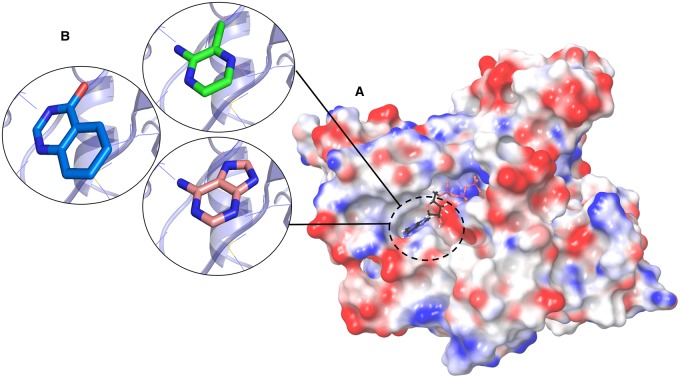
Targeting essential enzymes in *Mycobacterium abscessus*: Fragment hits for PurC (SAICAR Synthetase), an enzyme of *de novo* purine biosynthesis. (**A**) Surface electrostatic representation of PurC in complex with ATP. (**B**) Fragments occupying the adenylyl pocket of PurC making strong polar contacts. Figure prepared using PyMOL and Maestro (Schrödinger, LLC).

Fragment-based drug discovery has also been used to develop new antimicrobials, particularly for tuberculosis and to target other mycobacteria. Very little work is currently developed in big pharma against pathogens that are responsible for infectious disease in developing countries or those that cause disease to small sections of the population, for example by *Mycobacterium abscessus*, which causes life-threatening lung infections in cystic fibrosis patients [30]. Here, the fragment-based approach has the advantage of working with very small molecules that can penetrate the cell walls. Figure 1 illustrates the binding of fragments to an essential enzyme in *Mycobacterium abscessus*, a non-tuberculous Mycobacterium (Thomas, S.E., Mendes, V. and Blundell, T.L., unpublished data). Our screening data have shown that fragments can bind at allosteric and interfacial sites. The allosteric sites are often not identified as functional, but occasionally even these prove to have an impact on binding at the active site, as we have recently discovered with Mycobacterium tuberculosis CoaBC enzyme (Mendes, V., Blaszczyk, M. and Blundell, T.L., unpublished results).

## Mutations and disease

Many disease-related mutations, which are usually in residues that are conserved or conservatively varied during evolution, have an impact on the function of proteins. Classification and better interpretation of disease and non-disease-associated mutations is challenging but central to the understanding of genetic disease. Structural analyses of disease-causing mutations indicate that most occur at solvent-inaccessible and hydrogen-bonded residues [31].

Based on these observations, we proposed that the effect of a mutation on protein stability could be calculated by considering the statistical potential of a residue to change across evolution. The method site-directed mutator (SDM) uses a set of conformationally constrained, environment-specific substitution tables, which consider a residue's conformation, solvent accessibility and side-chain hydrogen bonding, to calculate the difference in stability between the wild-type and mutant protein structures [32,33]. More recently, we have used graph-based signatures to represent the wild-type structure environment in order to predict the effect of mutations on stability [34,35], and interactions with other proteins [35–39], nucleic acids [35], small molecules [40–42] and metal ions [43].

**Figure 2.**
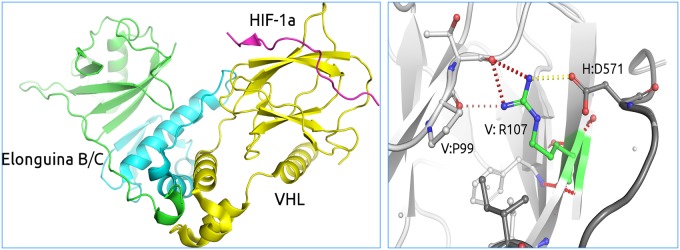
Genetic diseases mutations: clear cell renal carcinoma in von Hippel–Lindau disease. Left-hand side panel: the structure of the ternary complex of pVHL with Elongin C and Elongin B, critical for pVHL stability and function [50]. Right-hand side panel: Inter-subunit interactions mediated by arginine (R107). In VHL disease some mutations, for example at R107, alter the charge complementarity of the subunits and destabilise protein–protein interactions, essential to function.

These programs have provided insights into mutations that lead to a range of human genetic diseases, including cancers. For example, von Hippel–Lindau Syndrome leads to the development of clear cell renal carcinoma and is caused by mutations in the VHL gene (Figure 2). By considering the effect of the mutations upon the stability of the VHL protein and how they alter the affinity for its various binding partners, HIF-1α and Elongin B and C, we could accurately identify a patient's risk of developing clear cell renal carcinoma [43,44]. In subsequent studies looking at other human Mendelian diseases, we have consistently observed that mutations affecting protein stability represent the majority of disease mutations, but those that affect key protein–protein interactions have proved also to be common and important [45–48]. Interestingly, changes in protein stability have even been linked to population phenotypic variability, including drug responses [49].

The same molecular consequences also drive the development of drug resistance. Looking at single-point coding mutations in *Mycobacterium tuberculosis*, we observed strong correlations between the structural features of mutations and their link to antibiotic sensitivity [51]. This suggested the possibility that we could predict resistance mutations before they arise, allowing us to suggest optimal therapeutic interventions based on genomic sequences and guide drug development.

**Figure 3.**
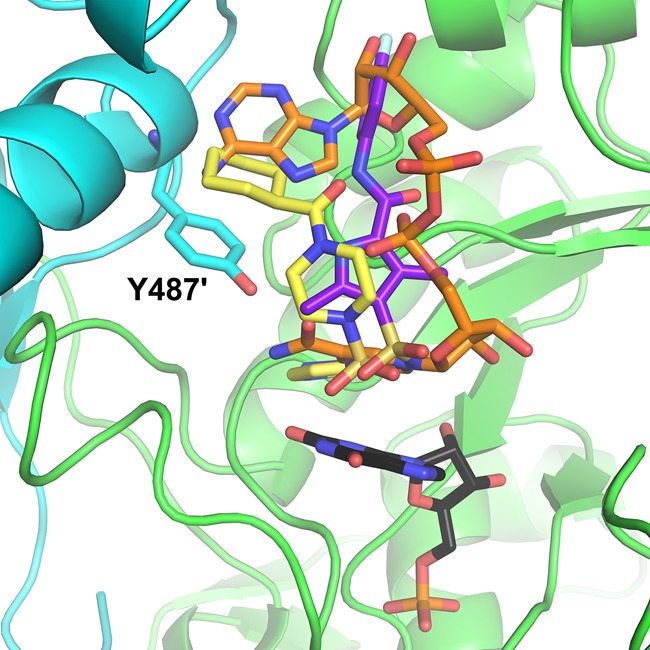
Avoiding resistance hotspots. The GuaB2 inhibitor VCC234718 (represented as yellow sticks [52]) stacks on top of IMP (represented as black sticks) in a similar manner to NAD (represented as orange sticks), but makes different interactions to the neighbouring protomer unit through Y487, which is mutated leading to resistance. Optimisation of a separate scaffold (Compound 6, represented as purple sticks [53]) more closely mimicked the interactions made by NAD, and was active against the Y487C mutant.

We have applied this theory to our recent efforts to develop small-molecule inhibitors of GuaB2 to treat *Mycobacterium tuberculosis.* Analysis of the crystal structure of GuaB2 with VCC234718 revealed the most likely resistance mutation would occur at Y487, altering interactions with the inhibitor but not NAD, while not disrupting protein stability or the interactions between the homo-tetramer units. Y487C was the main resistance mutation observed [52]. By modifying the inhibitor, altering the interactions being made, the resistance profile could be altered. Other molecules were identified that did not make these interactions and were active against the Y487C mutant [53] (Figure 3). While these inhibitors were shown to be active against Mtb *in vitro* through the inhibition of GuaB2, further work is needed to explore the potential therapeutic benefit of GuaB2 inhibition *in vivo*.

These analyses highlight the power of considering the structural environment of a mutation in order to understand the molecular and biological consequences. While many disease mutations are buried, simple classification of residues by solvent accessibility does not provide all of the information encompassed within the graph-based signatures, necessary to consider longer-range allosteric effects and to distinguish disease-causing mutations. This led us to consider other structural features to locate a residue within the interior of the structure.

## Refining our understanding of the impacts of mutations on protein function

The interiors of protein structures can be further classified using the conservation of residues in an orthologous family, as well as the packing density and the depth of the residue. These can provide more detailed information regarding the structural environment used in our substitution tables in SDM, allowing more accurate prediction of the effects of mutation and better identification of disease-related mutations.

We have recently analysed the residue side-chain relative solvent accessibility (RSA) [54], packing density, [55] and depth below the surface (http://cospi.iiserpune.ac.in/depth) [56,57] of residues across ∼15 000 high-resolution (≤2.5 Å), representative protein structures. This demonstrated that solvent-inaccessible residues (RSA <7%) exhibit varying levels of packing, with those closer to the core of the protein being more tightly packed. This suggests that further categorisation<UK ise of the environment-specific substitution tables in SDM with respect to depth and packing density might be useful in the prediction of the impact of mutations on protein stability [32].

**Figure 4.**
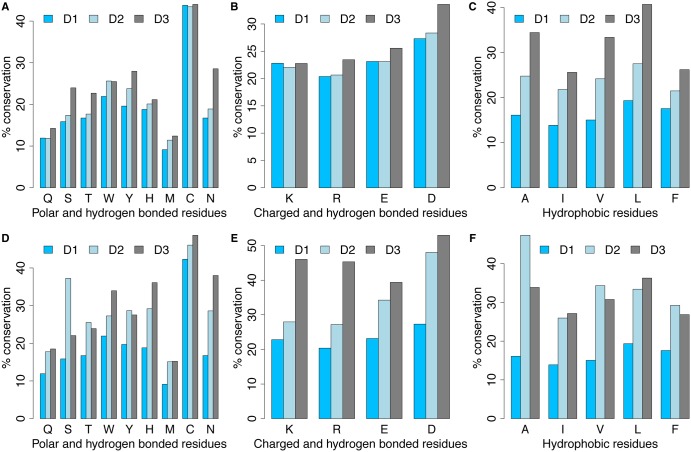
Residue conservation as a function of residue packing (A–C) and depth (D–F). D1 corresponds to all residues with RSA > 7%. The remaining inaccessible residues were categorised into the D2 and D3. For the case of packing (**A**–**C**), D2 and D3 corresponds to residue packing values ≤0.56 and >0.56, respectively. For depth (**D**–**F**), D2 and D3 corresponds to residue depth values ≤6 and >6 Å, respectively. Residue conservation with packing and depth were analysed for residues grouped on the basis of polar, charged and hydrophobic properties. Residue conservations were obtained from the substitution tables calculated using the ULLA program [58]. Ulla uses structural alignment of protein families annotated by the program JOY, indicating the local conformation and side-chain environment [54] for the purpose of calculating substitution tables.

We have found that residue conservation progressively increases as a function of residue packing, but the dependence on depth is more residue-specific (Figure 4; Pandurangan, A.P., work in progress). We are now modifying the environment-specific substitution tables to reflect these observations. Both these structural features could also add useful information while training models using machine-learning approaches to predict the impact of mutations on protein stability and affinity.

We have also asked whether we could better identify disease-causing mutations based on residue depth, packing density and conservation scores of the protein orthologues. To test this idea, we have investigated mutations occurring in alkaptonuria (AKU), which was the first characterised human Mendelian disease and is also known as black bone disease. AKU is caused by mutations in homogentisate-1,2-dioxygenase (HGD; PDB ID: 1EY2) that lead to the accumulation of reactive metabolites. There are over 80 known single-point mutations in HGD that have been linked to the development of AKU. We have previously shown that these mutations affect the activity of the protein complex primarily through destabilisation of the individual protomer structure but also through disruption of protomer–protomer interactions within the hexamer [45,46].

**Figure 5.**
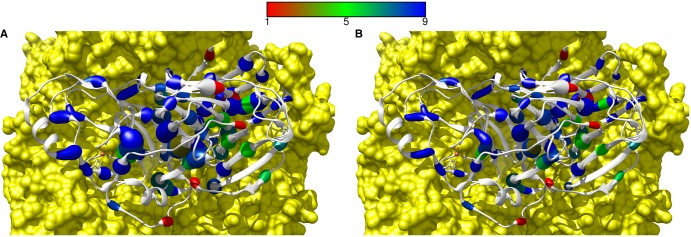
Mapping of residue conservation along with (A) residue packing and (B) residue depth for disease-related mutations in AKU. In (**A**) and (**B**), one of the protomers is shown as a ribbon diagram and the remaining five protomers are shown as a surface representation coloured in yellow. The disease mutants are coloured based on residue conservation. The conservation score range between 1 and 9 with low (in red) and high (in blue) score values corresponding to low and high conservation, respectively. In (**A**), the increasing thickness of the ribbon corresponds to the increasing residue packing in the protomer. Similarly, in (**B**), the increasing thickness of the ribbon corresponds to the increasing residue depth in the protomer. The colour gradient bar representing the residue conservation score is shown on top of the figure.

To visualise the relationship between residue conservation, depth and packing, we mapped the respective properties onto the structure of HGD (Figure 5). The residue conservation score was calculated using ConSurf [59]. In AKU, about 70% of the disease mutants are highly conserved (conservation scores >7), whereas for non-disease residues only 36% are highly conserved. Disease mutants proximal to the interface and the iron-binding site are well conserved for the purpose of maintaining interaction and function, respectively. In general, residue conservation could act as a potential indication to distinguish between disease mutant and non-disease residues.

To better dissect the properties unique to disease mutants, we looked at the correlation between depth and conservation for disease mutants and non-disease residues. As expected, depth was strongly correlated with conservation for both disease mutants and non-disease residues. For disease-causing mutants, the correlation between packing and conservation was significantly higher compared to the non-disease residues in AKU. Residue conservations have been previously shown to be greater than expected from analysis of amino acid, environment-dependent substitution tables in residues that are closer to functional binding sites [60], and have been shown to be dependent on the distance of the residue from the catalytic site [60,61].

This suggests that residue packing, depth and conservation are crucial parameters for the understanding of evolutionary constraints in protein structure and have potential applications in predicting the impact of mutations on protein stability and interactions. While residue depth is well correlated with residue conservation, the residue packing density offers a greater potential to differentiate between disease and non-disease mutations. We are now investigating the impact of water molecules that are bound in the interior and are completely solvent-inaccessible in order to further refine the calculation of packing densities.

In many cases multiple mutations have impacts on resistance, some increasing the resistance and others compensating for the original mutation. One approach is to use SDM or mCSM (see above) to estimate whether a single mutation is stabilising or destabilising. We model this mutation into the protein and treat it as the wild-type. We can then explore whether further mutations are likely to be stabilising, compensating for the first or lead to further instability. In future, we intend to look at the impacts of multiple mutations using machine-learning methods.

## Future challenges and applications of structure-guided drug discovery

Structure-guided approaches have demonstrated clearly that they can contribute to many aspects of early drug discovery, both computational and experimental. Structure can also inform our understanding of impacts of mutations in human genetic diseases and drug resistance in cancers and infectious disease. The Open Source Drug Discovery programme in India (http://www.osdd.net/about-us) has developed approaches using structural information to identify current, off-licence drugs that might be repurposed for use against infectious diseases in developing countries.

Our and others' research on understanding the impacts of mutations in genetic disease has demonstrated that mechanisms often involve interfacial disruptions and allosteric impacts on multiprotein assemblies and more simple oligomeric protein functions. Here, the design of interfacial stabilisers offers an attractive way forward that depends on a detailed knowledge of protein structure. Other approaches to combatting resistance include the use of peptidic inhibitors, which have more intrinsic flexibility and can often adopt other conformations to make alternative interactions with the mutant protein.

Our analysis of mechanisms of emergence of resistance in cancer and infectious disease and the methods for predicting it have been equally dependent on structural insights on protein targets. In combating resistance, an initial objective might be to design the candidate drug to interact only with residues used by the natural ligands; this would increase the chance that mutations affect the viability of the tumour or infectious agent as well as the drug. However, if it is necessary to find drug interactions beyond the interaction site of the natural substrates and cofactors, *in silico* saturation mutagenesis can be used to inform the choice of ways to grow fragments or redesign drugs, in such a way that they would less likely give rise to resistance mutations. In all these ways structural biology appears to be a central component of future efforts in drug discovery!

## Abbreviations

AKU: alkaptonuria
HGD: homogentisate-1,2-dioxygenase
NMR: nuclear magnetic resonance
RSA: relative solvent accessibility
SDM: Site-Directed Mutator
SPR: surface plasmon resonance.
